# Automation for lateral flow rapid tests: Protocol for an open-source fluid handler and applications to dengue and African swine fever tests

**DOI:** 10.1371/journal.pgph.0002625

**Published:** 2024-11-25

**Authors:** Rohan Laurent, Benjamin Hinnant, Michael D. Talbott, Kenneth Kim

**Affiliations:** 1 Histopathology Core, La Jolla Institute for Immunology, La Jolla, California, United States of America; 2 La Jolla Institute for Immunology, La Jolla, California, United States of America; Aga Khan University, PAKISTAN

## Abstract

Lateral flow rapid diagnostic tests (RDTs, RTs) are cost-effective with low infrastructure requirements for limited-resource settings, and in any setting can represent a bridge between early disease monitoring at outbreak onset and fully-scaled molecular testing for human or animal diseases. However, the potential of RTs to handle higher throughput testing is hampered by the need for manual processing. Here we review dengue virus and African swine fever virus rapid tests, and present a novel protocol that employs an open-source fluid handler to automate the execution of up to 42 RTs per run. A publicly accessible website, rtWIZARD.lji.org, provides printouts for correctly spacing cassettes, worksheets for sample organization, and test-specific fluid handler protocols to accurately deliver samples from a 48-tube rack to each cassette’s sample and running buffer wells. An optional QR-coded sheet allows for de-identified sample-to-result traceability by producing a unique printable label for each cassette, enabling results to be entered via a scanner. This work describes a highly cost-effective model for increasing outbreak diagnostic efficiency and of increasing RT throughput for other applications including workplace testing, food safety, environmental testing, and defense applications.

## Introduction

From a One Health perspective, the period from 2018 to the present has culminated in ongoing simultaneous, millions-scale outbreaks of four seemingly unrelated viral diseases: a novel genotype of African Swine Fever Virus (ASFV), a double-stranded DNA virus spread by fomites and the sole member of family Asfarviridae, was detected in August 2018 and has since resulted in the loss of over 40 million swine in Eurasian countries [1, 2]. Dengue virus (DENV), a mosquito-borne single (+) stranded RNA virus in family Flaviviridae, has been known to circulate through tropical and sub-tropical regions since its discovery in 1943 with a cycle period of approximately 3–5 years. However, in 2019 a massive outbreak in southeast Asia saw case numbers jump by 46% compared to 2015 [3], and globally it is estimated that over 9.5 million people were infected with DENV from 2019–2022 [4]. These case numbers do not account for the current unprecedented DENV outbreak in the Americas, which has resulted in over 4.6 infections in 2023 and 9.7 million infections the first half of 2024 alone [5, 6]. The H5N1 highly pathogenic avian influenza virus, a single (-) stranded RNA virus belonging to family Orthomyxoviridae, emerged in wild birds in 2020 and has since resulted in the culling of over 130 million poultry [7, 8]. The H5N1 virus has also been shown to infect over 30 species of mammals including humans [9–11]. Fourthly, severe acute respiratory syndrome coronavirus 2 (SARS-CoV-2), a single (+) stranded RNA virus belonging to family Coronaviridae, was first detected in December 2019 and has tragically caused the death of close to 7 million people [12]. SARS-CoV-2 also infects dozens of mammalian species [13].

From an economic perspective, although comprehensive data on the global economic impacts of these simultaneous outbreaks are not available, limited data indicate massive impacts. For example, it is estimated that the ASFV outbreak in China has resulted in a 1.4% to 2.07% decline in the country’s GDP of $17tn [2]. In 2016 it was estimated that the total annual global cost resulting from dengue illness was $8.9bn [14]. United States losses from the H5N1 outbreak in 2022 were estimated at over $2bn [15, 16]. These losses include the depopulation of over 43 million egg-laying hens, resulting in a 210% year-over-year increase in egg prices by the end of 2022 [17]. Finally, the most recent estimates indicate that at the end of 2023, the COVID-19 pandemic resulted in $14tn worth of losses for the US economy [18–20].

For many countries, the COVID-19 pandemic was a stark reminder that management and measurement of infectious disease outbreaks is entirely dependent on diagnostic testing [21]. Reverse-transcription quantitative polymerase chain reaction (RT-qPCR) remains the gold standard for infectious agent identification, and COVID-19 has fueled a next generation digital PCR revolution [22]. However, 18 years ago Yager et al. insightfully remarked that these gold standard tests, despite their usefulness, “were designed for air-conditioned laboratories, refrigerated storage of chemicals, a constant supply of calibrators and reagents, stable electrical power, highly trained personnel and rapid transportation of samples” [23]. Ironically, the countries and settings which stand to be the most impacted by infectious disease outbreaks are often the same areas which possess the least resources to test for infectious disease [24]. While each low diagnostic resource setting results from its own unique combination of contributing factors including laboratory infrastructure, distance to laboratory, equipment, personnel, and regulatory restrictions, the final measure of diagnostic efficacy in any setting can be described in just eight words: turnaround time for a quality and affordable test [25].

Lateral flow rapid diagnostic tests (RDTs, RTs) are cost-effective with low infrastructure requirements for low-resource settings, and in any setting can represent a bridge between outbreak onset and fully-scaled molecular testing for human or animal diseases. RTs are also utilized for other applications including workplace testing [26], food safety [27, 28], environmental testing [29–33], and defense applications [34]. However, the potential of RTs to handle higher throughput testing is hampered by the need for manual processing. Here we present a novel *rtWIZARD* protocol that employs an open-source fluid handler to automate the execution of 42 RTs from blood samples in 1.1ml or 2ml tubes. To demonstrate the relevance of the protocol to RTs in current utilization, we first review RTs for DENV and ASFV evaluated by the World Health Organization and the World Organisation for Animal Health, respectively. Protocol users download fluid handler scripts, printouts for correctly spacing cassettes, and optional printable QR-coded labels from a publicly accessible website, rtWIZARD.lji.org. QR codes can ensure de-identified sample-to-result traceability and enable results to be entered via a scanner. This work presents a model and protocol for automated performance of RTs.

## Dengue virus rapid tests

Most RTs for DENV target IgM and IgG against DENV nonstructural protein 1 (NS1). It should be noted that there are four serotypes of DENV, and that assay performance varies with outbreak serotype [35–40]. In 2009 the World Health Organization (WHO) reviewed three commercially available DENV antibody RTs [41], and a summary of these tests is provided in **Table 1** [42, 43]. A 2018 study indicates that the WHO has approved the use of a DENV NS1 antigen RT for diagnosis of DENV [44], but we are unable to confirm this statement [45]. No RTs were listed in the 2024 update of the WHO Prequalified In Vitro Diagnostics test list [46]. In 2018 the US Food and Drug Administration (FDA) cleared the InBios DENV Detect^TM^ NS1 ELISA Kit [45]. InBios also produces the Dengue NS1 Detect Rapid Test, but this strip test (without cassette) is not FDA cleared. In 2021, the United States Centers for Disease Control and Prevention (USCDC) adopted the CTK BIOTECH OnSite Dengue IgG Rapid Test as part of a two-test protocol to determine convalescence from prior DENV infection [47]; information on this test is also provided in **Table 1**. It should be noted that the OnSite Dengue IgG Rapid Test is a different test with differing performance characteristics from the OnSite Dengue IgG/IgM Combo Rapid Test. Overall, there is no RT for diagnosis of acute DENV infection authorized by the USCDC or the European Centre for Disease Prevention and Control. While there are several *conformité européenne*(cϵ)-marked tests for DENV, cϵ marking is not an indicator of regulatory approval or test quality.

**Table 1:** Select rapid tests for DENV infection [41].

Test name	Catalog no.	Manufacturer	Country	Target	Specimen type(s)	Storage	Testing time	Sensitivity	Specificity	Lysis step?	Running buffer	rtWIZARD compatible?	Refs.
**Panbio Dengue Duo Cassette**	01PF10	Abbott	Australia	IgM and IgG against NS1	Whole blood, plasma, serum	2-30 C	15min	77.80%	90.60%	No	2 drops	Yes	41
**Bioline Dengue IgG/IgM**	11FK10	Abbott	S. Korea	IgM and IgG against E protein antigen	Plasma, serum	1-30 C	15-20min	60.90%	90.00%	No	4 drops	Yes	41
**Dengucheck-WB**	NA	Zephyr Biomedicals	India	IgM and IgG against NS1	Whole blood, plasma, serum	4-30 C	15min	20.50%	86.70%	No	5 drops	Yes	41
**OnSite Dengue IgG Rapid Test **	NA	CTK BIOTECH	USA	IgG against envelope antigens from DENV1-4	Whole blood, plasma, serum	2-30 C, control samples at 2-8 C	15min	91.1-95.3%	92.8-98%	No	1 drop	Yes	42, 43

## African swine fever rapid tests

At the time of this publication, there is no RT for ASFV approved by the United States Department of Agriculture or European Union Reference Laboratory for ASF. In 2022 the World Organisation for Animal Health (OIE) reviewed three RTs for ASFV antigen testing and three RTs for ASFV antibody testing [48]. While no RTs are listed for diagnostic use in OIE’s *Terrestrial Manual* [49], the OIE review acknowledged that point of care tests “are a very useful adjunct to, but not a replacement for, laboratory testing in ASFV disease control programmes” [48]. A list of the ASFV antigen and antibody tests reviewed by OIE is provided in **Table 2** [50–53].

**Table 2:** World Organisation for Animal Health (OIE)-evaluated rapid tests for ASFV infection [48].

Test name	Catalog no.	Manufacturer	Country	Target	Specimen type(s)	Storage	Testing time	Sensitivity	Specificity	Lysis step?	Running buffer	rtWIZARD compatible?	Refs.
**Ingezim ASF CROM Ag**	11.ASFV.K.42	Gold Standard Diagnostics	Spain	Antigen VP72	Whole blood	2-8 C	15min	68%	98%	No	3 drops	Yes	50
**Rapid ASFV Ag**	RG1407DD	Bionote	S. Korea	Antigens VP72 and VP32	Whole blood, plasma, serum	2-30 C	20min	Low to moderate	Moderate	No	None	Yes	NA
**SLB ASF Antigen Detection RDT**	NA	Shenzhen Lvshiyuan Biotechnology Co.	China	Antigen undisclosed	Whole blood	2-30 C	15-20min	65%	76%	Yes	None	No	51
**Ingezim ASFV-CSFV CROM Ab**	11.SFV.K41	Gold Standard Diagnostics	Spain	Antibody against VP72	Whole blood, plasma, serum	4-25 C	15-30min	87%	100%	NA	Yes	Yes	52
**Ingezim PPA CROM**	11.PPA.K41	Gold Standard Diagnostics	Spain	Antibody against VP72	Whole blood, plasma, serum	4-25 C	15-30min	82-99%	96-99%	No	3 drops	Yes	53
**GDX70-2 Herdscreen ASF Antibody**	NA	Global DX	UK	Antibody against VP30	Whole blood, plasma, serum	NA	15-30min	86-100%	100%	NA	NA	Yes	NA

## Materials and methods

The protocol described in this peer-reviewed article is published on protocols.io, https://dx.doi.org/10.17504/protocols.io.6qpvr8813lmk/v1, and is included for printing as **S1 Protocol** with this article.

### Objective

To automate the sample and running buffer dispensing process for rapid tests using the Opentrons OT-2 robotic liquid handler.

### Application of the protocol

While the rtWIZARD protocol can be used to automate virtually any cassette-based rapid test(RT), two specific use cases are RTs for dengue virus and African swine fever rapid tests. Dengue virus RTs include the Panbio Dengue Duo Cassette, Bioline Dengue IgG/IgM, Dengucheck-WB, and OnSite Dengue IgG Rapid Test. African swine fever RTs include the Ingezim ASF CROM Ag, Rapid ASFV Ag, Ingezim ASFV-CSFV CROM Ab, Ingezim PPA CROM and GDX70-2 Herdscreen ASF Antibody.

### Safety precautions

- Follow all safety and biohazard regulations in your jurisdiction.
- Handle samples with care, wearing appropriate PPE, including gloves, safety glasses, and lab coats.
- Dispose of used pipette tips and cassettes in a biohazard waste bin.
- After procedures, disinfect all equipment surfaces.
- If using a combustion inverter generator, follow manufacturer’s instructions regarding ventilation and safe operating distance.

### Equipment

**Fig 1:**
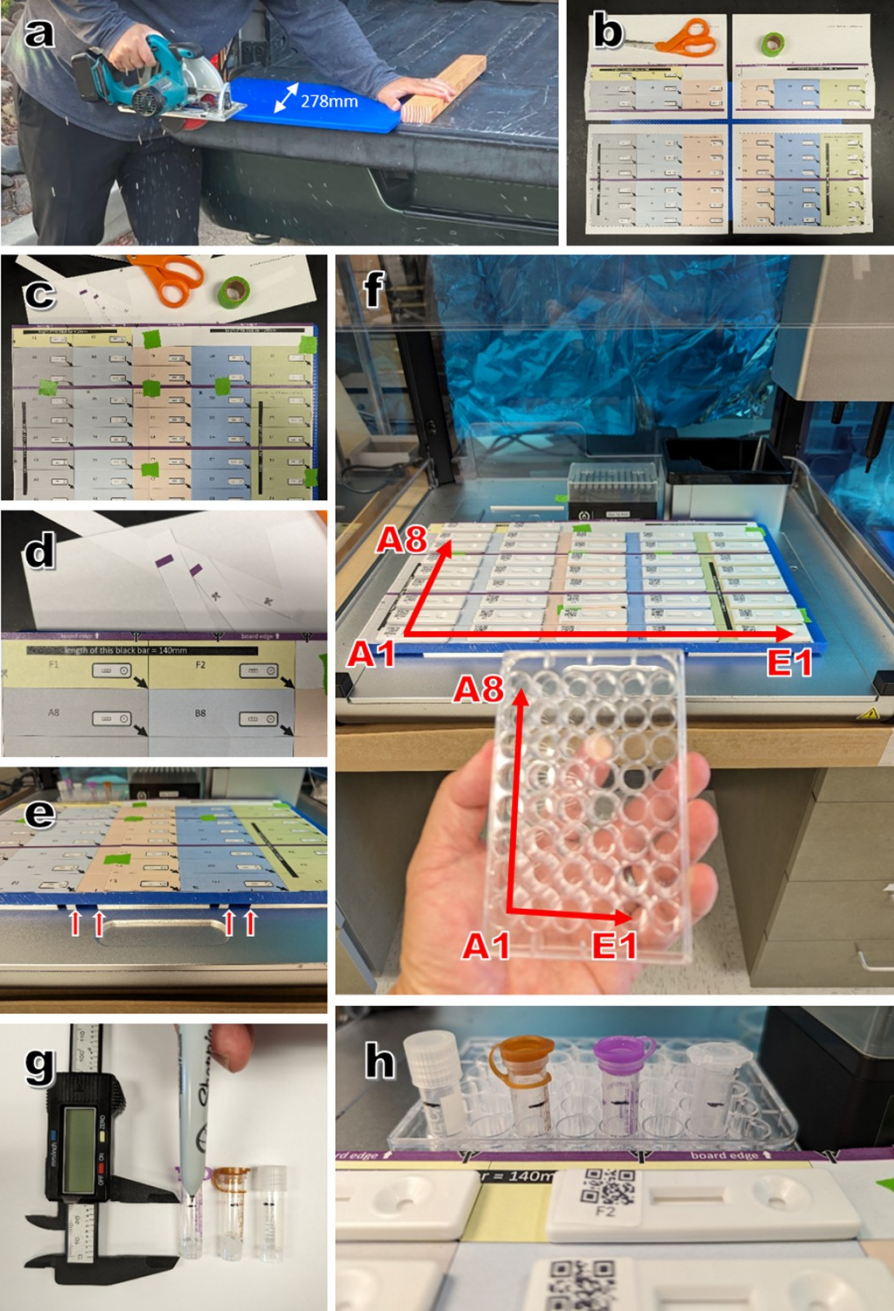
Preparation of rtWIZARD deck board, alignment of the cassette grid, and sample tube preparation. (a) The board can be 45.5–47.5cm long but should be 278mm wide. (b) The deck printout download produces four separate sheets for stitching. (c) Deck printout sheets stitched together into a grid with green tape. (d) The back edge of the grid should align with the back edge of the board. (e) Arrows at the front edge of the grid align with edges of retainer bars on the OT-2 deck. (f) Grid positions mirror well plate positions when the well plate is turned 90 degrees counterclockwise. (g) Sample tubes are marked with a line 25mm above the tube bottom. (h) Diverse 1.1-2ml tubes have bottom diameters of less than 11mm and thus will fit into wells of the 48 well plate.

- Opentrons OT-2. Because RTs utilize relatively crude pipetting, these tests are robust enough to tolerate small variations in micropipette performance. However, it should be noted that Opentrons recommends the OT-2 be used within specific ambient parameters (16-24C, humidity < 80%). If the OT-2 is used outside these parameters or in the field, the authors recommend that in between usages the OT-2 be stored in a desiccant box utilizing one of the solutions suggested in **S5 Appendix**, or in an air-conditioned environment.
- Opentrons P300 GEN2 Pipette (attached to OT-2 in Opentrons App)
- Computer and operating system compatible with Opentrons App
- Office printer
- Metric ruler or caliper
- Masking or laboratory tape
- Board. Non-porous material is preferred for cleaning, but plywood will suffice. Length: 45.5–47.5cm or 18–18¾ inches. Width: should be 278mm or board should be cut to this width (**Fig 1A**). Thickness: approximately 10mm or 3/8 inch.
- Scissors
- 2.5mm and 3.0mm hex screwdrivers (delivered with OT-2)
- Microcentrifuge for assays requiring plasma or serum
- Optional: inverter generator or power station rated for at least 500W
- Extension cord
- Optional: QR code scanner, laser printer
- Optional: The OT-2 ships with three locking brackets held in place by 3mm hex screws. For transport/field scenarios, it is recommended that these brackets be re-installed and removed at the testing location.

### Supplies

- Corning 48-well plate (Cat #3548) or any 48-well plate with well bottom diameter of 11mm or greater [acting as a rack for 42 sample tubes and 6 running buffer tubes]
- Up to 42 rapid tests
- Rack of Opentrons OT-2 Pipette Tips, 300μL, with at least 43 tips
- Indelible lab marker
- Translucent or clear sample tubes (1.1-2ml each) of less than 11mm bottom diameter
- 2ml running buffer tubes (if test utilizes running buffer) of less than 11mm bottom diameter
- Optional: 0.75 x 0.75” printable labels (Avery 94102)

### Downloads

- Opentrons App
- From rtWIZARD.LJI.ORG:
  ○ Deck printouts (**S1 Appendix**)
  ○ Test-specific rtWIZARD Opentrons protocol (import into Opentrons App)
  ○ Test-specific rtWIZARD Opentrons labware definitions (import into Opentrons App)
  ○ rtWIZARD manual plate record (**S2 Appendix, S3 Appendix**)
  ○ Optional: rtWIZARD plate record spreadsheet

### Procedure

**Fig 2:**
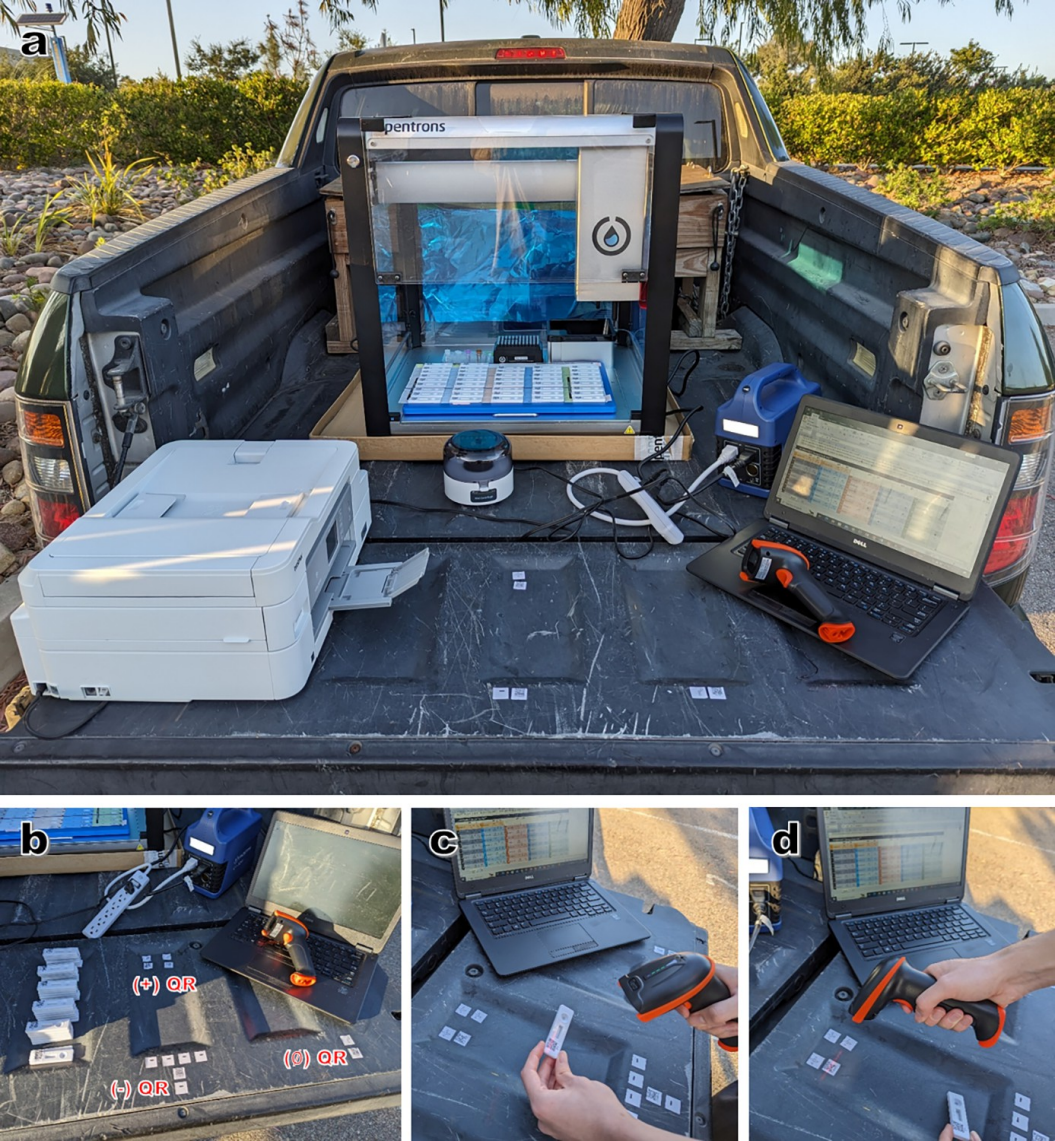
Embodiment of field testing station for use of rtWIZARD protocol. (a) A portable power bank can power several pieces of equipment including the OT-2, laptop computer, a centrifuge, an office printer, and lights. (b) The rtWIZARD QR code sheet includes codes for resulting by scanner. The rtWIZARD plate record spreadsheet includes (c) a script/macro for automatic cursor movement and (d) these cells can be populated by scanner as well.

**1. Testing grid and board preparation**
1.1 Print the four pages in the deck printouts file (**Fig 1B**).
1.2 Using ruler, measure the 140mm scale bar on grid printouts to verify that dimensions are true. If dimensions need adjusting, adjust scale in printer settings until dimensions are true.
1.3 Using adhesive tape, stitch the four printouts (**Fig 1C**). Proper sheet alignment can be assured by viewing backlighting (overhead lights, lamp, sunlight) through two overlapping sheets.
1.4 Following grid assembly, align the edge of the rear bar to the rear edge of the board (**Fig 1D**) and tape onto the board.
**2. Aligning grid with OT-2 deck**
2.1 Seat the 48-well plate in bay 10 and the pipette tip box in bay 11
2.2 Place the board over bays 1–9 and push back until the rear of the board is sitting flush with the pipette tip box and the 48-well plate.
2.3 See the small arrows at the front of the grid/board, and make sure these arrows align with the ends of the retainer bars under the board (**Fig 1E**).
**3. Sample collection**
3.1 Prior to sample collection, use marker to draw a horizontal line 25mm from the bottom of each tube (**Fig 1G and 1H**). This will ensure that the pipette tip is submersed in sample.
3.2 User marker to label tube with sample ID.
3.3 At collection, fill tube to 25mm line or higher.
3.4 Optional: If sample sources are known prior to collection, identities can be entered into plate record spreadsheet.
**4. Well plate (rack) organization**
4.1 Complete manual plate record, assigning each sample to a plate position and writing the plate position on the sample tube. Assign samples in order of columns from wells A1 to F2.
4.2 Centrifuge samples for RTs requiring serum or plasma.
4.3 Remove sample tube caps and place each tube in its assigned well. For tubes with flip-top lids, remove the lid by either sliding it off or cutting the lid hinge.
4.4 For RTs requiring running buffer, expel running buffer from each test kit into clean 2ml tubes. As each tube is filled, place the tube in wells F3-F8. RT kits routinely provide running buffer volumes in excess of amount required for tests. For 42 tests, RTs requiring:
  - 1 standard drop of running buffer per test will require 1.5 tubes filled in F3-F4.
  - 2 standard drops of running buffer per test will require 2.5 tubes filled in F3-F5.
  - 3 standard drops of running buffer per test will require 3.5 tubes filled in F3-F6.
  - 4 standard drops of running buffer per test will require 4.5 tubes filled in F3-F7.
  - 5 standard drops of running buffer per test will require 5.5 tubes filled in F3-F8.
4.5 Place well plate in OT-2 bay 10.
4.6 Place pipette tip rack in OT-2 bay 11.
**5. Testing board organization**
5.1 Remove 42 RT cassettes from their packaging.
5.2 Holding each cassette with the sample and running buffer wells on the right, write the grid position on each cassette. Grid positions mirror well plate positions when the well plate is turned 90 degrees counterclockwise (**Fig 1F**).
5.3 Starting at the back row, place the cassettes in each cell. For each test justify the bottom right hand corner of the cassette to the bottom right hand corner of the cell.
5.4 Optional: Print QR code download (**S4 Appendix**) and create cassette labels with QR code and grid position.
**6. Use the Opentrons app to run the fluid handling protocol**
6.1 Note the time at which all drops of sample and running buffer were delivered to well A1.
**7. Resulting**
7.1 After the appropriate amount of incubation time, starting at the front row, record the results of each test by circling +,—or ∅. If the control line and target line show clear signal, then the test is positive(+). If the control line shows signal but the target line shows no signal, then the test is negative(-). If the control line shows no signal or any of the lines show ambiguous signal, then the test result is indeterminate (∅).
7.2 Optional: Open the spreadsheet in Microsoft Excel. Enable macros. Using the barcode scanner, scan each cassette and then the result (positive, negative, indeterminate) from the printed test menu (**Fig 2B–2D**). The scanned results will automatically populate into the results field.

## Results

To determine the efficiency of the rtWIZARD protocol, we performed trial runs using the Opentrons OT-2. Results are displayed in **Table 3**. Regarding individual cassettes, the time from sample delivery to running buffer delivery ranged from 261 seconds for the first cassette to 404 seconds for the 42nd cassette. Regarding the entire testing board of 42 cassettes, the mean time from the start of the protocol to the delivery of the last running buffer for the entire board of 42 cassettes was 733 seconds (12 minutes and 13 seconds) with a standard deviation of ± 22 seconds. In comparison, a laboratory technician provided with the same rack of 42 samples tubes and 8 running buffer tubes would have to deliver sample and running buffer to all cassettes at a rate of 733 seconds ÷ 42 cassettes = 18 seconds/cassette to match the efficiency of rtWIZARD. During this time the technician would be expected to make no errors such as sample-cassette mismatch, sample spillage, and cassette contamination. Additionally, in scenarios where 100 or more rapid tests are performed during a work shift, the technician would be required to maintain this efficiency to match the precision and efficiency of rtWIZARD.

**Table 3:** rtWIZARD protocol trial runs.

	Total run	Time between sample and buffer delivery (s)	
Trial	time (s)	1st cassette	42nd cassette
1	709	265	404
2	767	261	393
3	738	263	385
4	717	263	388
Mean ± SD	733 ± 22	263 ± 1	393 ± 7

## Discussion

The current simultaneous and ongoing large-scale outbreaks of human and animal diseases, along with their economic consequences, underscore the vital importance of rapid, reliable, and scalable diagnostic tests. While RT-qPCR remains the gold standard, PCR is not always feasible at the onset of an outbreak or in settings without proper resources, such as a nearby laboratory or trained personnel. Though imperfect, especially in respect to scaling, rapid tests have emerged as important tools due to their combination of simplicity, resilience to ambient conditions, cost, improving quality, and lack of infrastructure and personnel requirements. Our introduction of the rtWIZARD protocol provides moderate scaling of RTs at a cost that is affordable to many but not all organizations. The throughput benefit afforded by rtWIZARD results from batching and requires not inconsiderable pre-analytic sample organization; thus the rtWIZARD model is not suited for individual urgent bedside testing.

Prior to the COVID-19 pandemic, the RT market had an anticipated value of $8.2 billion by 2022 [54]. In the short span of six months (Q4 2020—Q1 2021), just three major US manufacturers (Abbot, Quidel, and Becton Dickinson) accounted for sales exceeding $5.5 billion. Such growth of the RT market points towards a bolstering of RT research and development, manufacturing capacity and supply chains. Grant funding initiatives, like NIH RADx [55], are providing further financial and other resources to foster innovation and increase RT production. Consequently, the RT market is poised for further expansion in the near- to medium-term [54].

While we present the rtWIZARD model as a bridge solution for African swine fever or dengue virus outbreaks, the protocol can be easily modified for more routine testing scenarios in which large numbers of RTs are performed. Examples of other testing scenarios include pregnancy testing, workplace testing, food safety, environmental testing, and defense applications. When applied properly, rtWIZARD can provide faster, cheaper, and better information for One Health and other applications.

### Limitations

This work describes a model for increasing outbreak diagnostic efficiency and of increasing RT throughput for other applications. Sites using these protocols should verify it locally for test accuracy, competence of staff and efficiency. For research use only.

## Supporting information

S1 Protocol rtWIZARD protocol with the Opentrons OT-2v1. (PDF)

S1 Appendix rtWIZARD deck printouts. (PDF)

S2 Appendix rtWIZARD manual plate record, one_analyte. (PDF)

S3 Appendix rtWIZARD manual plate record, two analyte. (PDF)

S4 Appendix rtWIZARD QR codes for Avery 94102. (PDF)

S5 Appendix Commercial suppliers. (PDF)
