# Supplementary material for: Automation for lateral flow rapid tests: Protocol for an open-source fluid handler and applications to dengue and African swine fever tests
Source: PLOS Glob Public Health. 2024 Nov 25;4(11):e0002625. doi: 10.1371/journal.pgph.0002625 (PMC11588214; doi:10.1371/journal.pgph.0002625)
Supplement: S1 Protocol — (PDF) [file pgph.0002625.s001.pdf]

Jul 11, 2024

rtWIZARD Protocol with the Opentrons OT-2 v1

DOI
dx.doi.org/10.17504/protocols.io.6qpvr8813lmk/v1

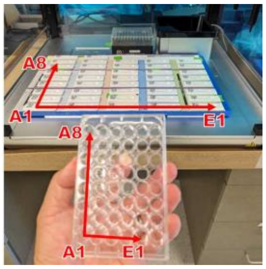

Rohan Laurent[1], Benjamin Hinnant[1], Michael D. Talbott[1], Kenneth Kim[1]
[1]La Jolla Institute for Immunology

Benjamin Thomas Hinnant
La Jolla Institute for Immunology

OPEN ACCESS

DOI: dx.doi.org/10.17504/protocols.io.6qpvr8813lmk/v1

External link: https://rtwizard.lji.org/

Protocol Citation: Rohan Laurent, Benjamin Hinnant, Michael D. Talbott, Kenneth Kim 2024. rtWIZARD Protocol with the Opentrons OT-2 v1. **protocols.io** https://dx.doi.org/10.17504/protocols.io.6qpvr8813lmk/v1

Manuscript citation:

Automation for Lateral Flow Rapid Tests: Protocol for an Open-Source Fluid Handler and Applications to Dengue and African Swine Fever Tests. (2024) Laurent, R; Hinnant, B; Talbott, MD; Kim, K.

License: This is an open access protocol distributed under the terms of the Creative Commons Attribution License, which permits unrestricted use, distribution, and reproduction in any medium, provided the original author and source are credited

Protocol status: In development
We are still developing and optimizing this protocol

Created: June 06, 2024

Last Modified: July 11, 2024

Protocol Integer ID: 101358

Keywords: Rapid diagnostic test, Rapid test, Lateral flow assay, Lateral flow immunoassay, Automation, Automate, Opentrons OT-2, Dengue virus, African swine fever virus

Funders Acknowledgement:

None

Disclaimer

For research use only.

Abstract

Starting with sample tubes racked in a 48-well plate, this rtWIZARD protocol uses the Opentrons OT-2 to automate performance of up to 42 rapid tests(RTs) or lateral flow assays. For single-well RTs, the expected total run time is less than 13 minutes. Macro-embedded spreadsheets and printable QR code labels are provided for ease of accessioning, sample organization, and resulting.

Attachments

Fig 1a.png
1.9MB

Fig 1b.png
1.9MB

Fig 1c.png
1.4MB

Fig 1d.png
2.1MB

Fig 1e-f.png

838KB

Fig 1g.png
1.1MB

Fig 2a-c.png
1.2MB

S2 Appendix 1 rtWIZA...
149KB

S3 Appendix 2 Manual...
153KB

S4 Appendix 3 Manual...
158KB

S5 Appendix 4 rtWIZA...
297KB

S6 Appendix 5 Commer...
121KB

Appendix 6 Manual Pl...
13KB

Appendix 7 Manual Pl...
13KB

Guidelines

For research use only.

Materials

Equipment

• Opentrons OT-2
• Opentrons P300 GEN2 Pipette (attached to OT-2 in Opentrons App)
• Computer and operating system compatible with Opentrons App
• Office printer
• Metric ruler or caliper
• Masking or laboratory tape
• Board. Non-porous material is preferred for cleaning, but plywood will suffice. Length: 45.5-47.5cm or 18-183⁄4 inches. Width: should be 278mm or board should be cut to this width (Fig 1a). Thickness: approximately 10mm or 3/8 inch.
• Scissors
• 2.5mm and 3.0mm hex screwdrivers (delivered with OT-2)
• Microcentrifuge for assays requiring plasma or serum
• Optional: inverter generator or power station rated for at least 500W
• Extension cord
• Optional: QR code scanner, laser printer
• Optional: The OT-2 ships with three locking brackets held in place by 3mm hex screws. For transport/field scenarios, it is recommended that these brackets be re-installed and removed at the testing location.

Supplies

• Corning 48-well plate (Cat #3548) or any 48-well plate with well bottom diameter of 11mm or greater [acting as a rack for sample and running buffer tubes]
• Up to 42 rapid tests
• Rack of Opentrons OT-2 Pipette Tips, 300µL, with at least 43 tips
• Indelible lab marker
• Translucent or clear sample tubes (1.1-2ml each) of less than 11mm bottom diameter
• 2ml running buffer tubes (if test utilizes running buffer) of less than 11mm bottom diameter
• Optional: 0.75 x 0.75” printable labels (Avery 94102)

Before start

· Follow all safety and biohazard regulations in your jurisdiction.
· Handle samples with care, wearing appropriate PPE, including gloves, safety glasses, and lab coats.
· Dispose of used pipette tips and cassettes in a biohazard waste bin.
· After procedures, disinfect all equipment surfaces.
· If using a combustion inverter generator, follow manufacturer’s instructions regarding ventilation and safe operating distance.

Testing grid and board preparation

1. Print the four pages in the deck printouts file (**Appendix 1, Fig 1a**).
2. Using ruler, measure the 140mm scale bar on grid printouts to verify that dimensions are true. If dimensions need adjusting, adjust scale in your printer settings until dimensions are true.

3. Using adhesive tape, stitch the four printouts (**Fig 1b**). Proper sheet alignment can be assured by viewing backlighting (overhead lights, lamp, sunlight) through two overlapping sheets.
4. Following grid assembly, align the edge of the rear bar to the rear edge of the board (**Fig 1c**) and tape onto the board.

Aligning grid with OT-2 deck

5. Seat the 48-well plate in bay 10 and the pipette tip box in bay 11.
6. Place the board over bays 1-9 and push back until the rear of the board is sitting flush with the pipette tip box and the 48-well plate.
7. See the small arrows at the front of the grid/board, and make sure these arrows align with the ends of the retainer bars under the board (**Fig 1d**).

Sample collection

8. Prior to sample collection, use marker to draw a horizontal line 25mm from the bottom of each tube (**Fig 1e-f**). This will ensure that the pipette tip is submersed in sample.
9. Use marker or printed QR code (**Appendix 4**) to label tube with sample identifier.
10. At collection, fill tube to 25mm line or higher.
11. Optional: If sample sources are known prior to collection, identities can be entered into plate record spreadsheet (**Appendices 6-7**).

Well plate (rack) organization

12. Complete manual plate record (**Appendices 2-3**), assigning each sample to a plate position and writing the plate position on the sample tube. Assign samples in order of columns from wells A1 to F2.
13. Centrifuge samples for RTs requiring serum or plasma.
14. Remove sample tube caps and place each tube in its assigned well. For tubes with flip-top lids, remove the lid by either sliding it off or cutting the lid hinge.
15. For RTs requiring running buffer, expel running buffer from each test kit into clean 2ml tubes. As each tube is filled, place the tube in wells F3-F8. RT kits routinely provide running buffer volumes in excess of amount required for tests. For 42 tests, RTs requiring:
    - 1 standard drop of running buffer per test will require 1.5 tubes filled in F3-F4.
    - 2 standard drops of running buffer per test will require 2.5 tubes filled in F3-F5.
    - 3 standard drops of running buffer per test will require 3.5 tubes filled in F3-F6.
    - 4 standard drops of running buffer per test will require 4.5 tubes filled in F3-F7.
    - 5 standard drops of running buffer per test will require 5.5 tubes filled in F3-F8.
16. Place well plate in OT-2 bay 10.
17. Place pipette tip rack in OT-2 bay 11.

Testing board organization

18. Remove 42 RT cassettes from their packaging.
19. Holding each cassette with the sample and running buffer wells on the right, write the grid position on each cassette. Grid positions mirror well plate positions when the well plate is turned 90 degrees counterclockwise (**Fig 1g**).
20. Starting at the back row, place the cassettes in each cell. For each test justify the bottom right hand corner of the cassette to the bottom right hand corner of the cell.

21. Optional: Print QR code download (**Appendix 4**) and create cassette labels with QR code and grid position.

Use the Opentrons app to run the fluid handling protocol

22. Note the time at which all drops of sample and running buffer were delivered to well A1.

Resulting

23. After the appropriate amount of incubation time, starting at the front row, record the results of each test by circling +, - or ∅. If the control line and target line show clear signal, then the test is positive(+). If the control line shows signal but the target line shows no signal, then the test is negative(-). If the control line shows no signal or any of the lines show ambiguous signal, then the test result is indeterminate (∅).
24. Optional: Open the spreadsheet plate record (**Appendices 6-7**) in Microsoft Excel. Enable macros. Using the barcode scanner, scan each cassette and then the result (positive, negative, indeterminate) from the printed test menu (**Fig 2a-c**). The scanned results will automatically populate into the results field.

Application of the protocol

25. While the rtWIZARD protocol can be used to automate virtually any cassette-based rapid test(RT), two specific use cases are RTs for dengue virus and African swine fever rapid tests. Dengue virus RTs include the Panbio Dengue Duo Cassette, Bioline Dengue IgG/IgM, Dengucheck-WB, and OnSite Dengue IgG Rapid Test. African swine fever RTs include the Ingezim ASF CROM Ag, Rapid ASFV Ag, Ingezim ASFV-CSFV CROM Ab, Ingezim PPA CROM and GDX70-2 Herdscreen ASF Antibody.

Protocol references

Hunsperger EA, Yoksan S, Buchy P, Nguyen VC, Sekaran SD, Enria DA, Pelegrino JL, Vázquez S, Artsob H, Drebot M, Gubler DJ, Halstead SB, Guzmán MG, Margolis HS, Nathanson C-M, Lic NRR, Bessoff KE, Kliks S, Peeling RW. Evaluation of Commercially Available Anti–Dengue Virus Immunoglobulin M Tests. Emerg Infect Dis. 2009;15:436–439.

Inui K, Gallardo C, Portugal R, Dixon L, Baton C, Williams D, Wang Z, Heath L, Sanchez-Vizcaino JM. The OIE ASF Reference Laboratory Network's overview of African swine fever diagnostic tests for field application. 2022;

Poltep K, Phadungsombat J, Kosoltanapiwat N, Hanboonkunupakarn B, Wiriyarat W, Suwanpakdee S, Prompiram P, Nakayama EE, Suzuki K, Iwamoto H, Shioda T, Leaungwutiwong P. Performance of the nonstructural 1 Antigen Rapid Test for detecting all four DENV serotypes in clinical specimens from Bangkok, Thailand. Virology Journal. 2022;19:169.

Prommool T, Sethanant P, Phaenthaisong N, Tangthawornchaikul N, Songjaeng A, Avirutnan P, Mairiang D, Luangaram P, Srisawat C, Kasinrerk W, Vasanawathana S, Sriruksa K, Limpitikul W, Malasit P, Puttikhunt C. High performance dengue virus antigen-based serotyping-NS1-ELISA (plus): A simple alternative approach to identify dengue virus serotypes in acute dengue specimens. PLoS Negl Trop Dis. 2021;15:e0009065.

Pollak NM, Olsson M, Ahmed M, Tan J, Lim G, Setoh YX, Wong JCC, Lai YL, Hobson-Peters J, Macdonald J, McMillan D. Rapid Diagnostic Tests for the Detection of the Four Dengue Virus Serotypes in Clinically Relevant Matrices. Microbiology Spectrum. 2023;11:e02796-22.

Lai S-C, Huang Y-Y, Wey J-J, Tsai M-H, Chen Y-L, Shu P-Y, Chang S-F, Hung Y-J, Hou J-N, Lin C-C. Development of Novel Dengue NS1 Multiplex Lateral Flow Immunoassay to Differentiate Serotypes in Serum of Acute Phase Patients and Infected Mosquitoes. Frontiers in Immunology [Internet]. 2022 [cited 2023 Sep 11];13. Available from: https://www.frontiersin.org/articles/10.3389/fimmu.2022.852452

Liu L-T, Chen C-H, Tsai C-Y, Lin P-C, Hsu M-C, Huang B-Y, Wang Y-H, Tsai J-J. Evaluation of rapid diagnostic tests to detect dengue virus infections in Taiwan. PLOS ONE. 2020;15:e0239710.

Santoso MS, Yohan B, Denis D, Hayati RF, Haryanto S, Trianty L, Noviyanti R, Hibberd ML, Sasmono RT. Diagnostic accuracy of 5 different brands of dengue virus non-structural protein 1 (NS1) antigen rapid diagnostic tests (RDT) in Indonesia. Diagnostic Microbiology and Infectious Disease. 2020;98:115116.

Sa-ngamuang C, Haddawy P, Luvira V, Piyaphanee W, Iamsirithaworn S, Lawpoolsri S. Accuracy of dengue clinical diagnosis with and without NS1 antigen rapid test: Comparison between human and Bayesian network model decision. PLoS Negl Trop Dis. 2018;12:e0006573.

Kabir MA, Zilouchian H, Younas MA, Asghar W. Dengue Detection: Advances in Diagnostic Tools from Conventional Technology to Point of Care. Biosensors (Basel). 2021;11:206.

Prequalified In Vitro Diagnostics | WHO - Prequalification of Medical Products (IVDs, Medicines, Vaccines and Immunization Devices, Vector Control) [Internet]. [cited 2024 Jul 10];Available from: https://extranet.who.int/prequal/vitro-diagnostics/prequalified-vitro-diagnostics

CDC. Pre-vaccination Testing | CDC [Internet]. Centers for Disease Control and Prevention. 2022 [cited 2023 Sep 5];Available from: https://www.cdc.gov/dengue/vaccine/hcp/testing.html

Liberal V, Forrat R, Zhang C, Pan C, Bonaparte M, Yin W, Zheng L, Viscardi V, Wu Y, Ataman-Önal Y, Savarino S, Chen C. Performance Evaluation of a Dengue IgG Rapid Diagnostic Test Designed to Determine Dengue Serostatus as Part of Prevaccination Screening. Microbiol Spectr. 10:e00711-21.

Savarino SJ, Bonaparte M, Wang H, Dayan GH, Forrat R, Zhu M, Hodge S, Ataman-Önal Y, DiazGranados CA. Accuracy and efficacy of pre-dengue vaccination screening for previous dengue infection with a new dengue rapid diagnostic test: a retrospective analysis of phase 3 efficacy trials. The Lancet Microbe. 2022;3:e427–e434.

Sastre P, Gallardo C, Monedero A, Ruiz T, Arias M, Sanz A, Rueda P. Development of a novel lateral flow assay for detection of African swine fever in blood. BMC Vet Res. 2016;12:206.

Matsumoto N, Siengsanan-Lamont J, Gleeson LJ, Douangngeun B, Theppangna W, Khounsy S, Phommachanh P, Halasa T, Bush RD, Blacksell SD. Evaluation of the diagnostic accuracy of an affordable rapid diagnostic test for African Swine Fever antigen detection in Lao People's Democratic Republic. J Virol Methods. 2020;286:113975.

Sastre P, Pérez T, Costa S, Yang X, Räber A, Blome S, Goller KV, Gallardo C, Tapia I, García J, Sanz A, Rueda P. Development of a duplex lateral flow assay for simultaneous detection of antibodies against African and Classical swine fever viruses. J Vet Diagn Invest. 2016;28:543–549.

Cappai S, Loi F, Coccollone A, Cocco M, Falconi C, Dettori G, Feliziani F, Sanna ML, Oggiano A, Rolesu S. Evaluation of a Commercial Field Test to Detect African Swine Fever. J Wildl Dis. 2017;53:602–606.
