# Supplementary figures and images for: Automation for lateral flow rapid tests: Protocol for an open-source fluid handler and applications to dengue and African swine fever tests
Source: PLOS Glob Public Health. 2024 Nov 25;4(11):e0002625. doi: 10.1371/journal.pgph.0002625 (PMC11588214; doi:10.1371/journal.pgph.0002625)

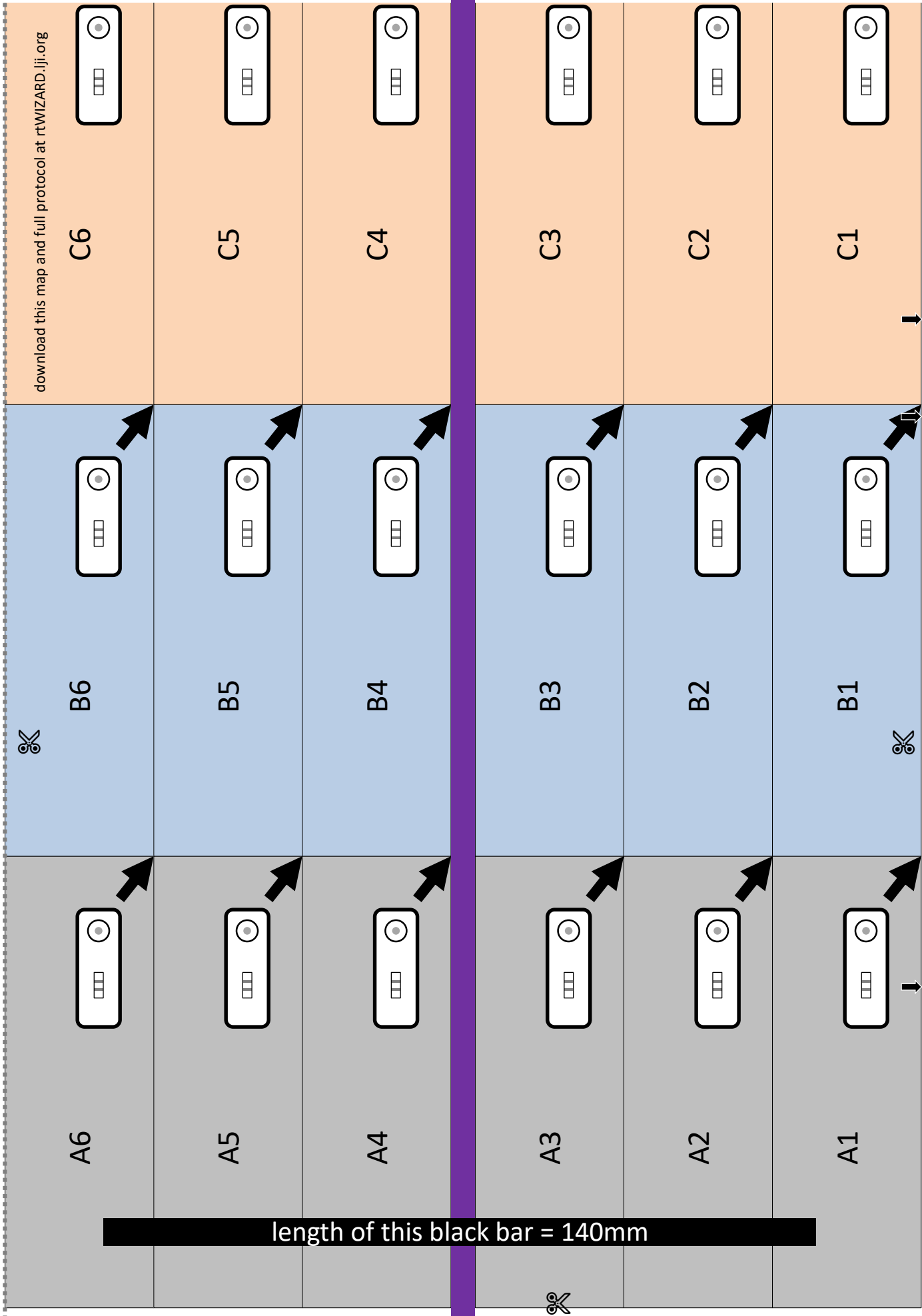

download this map and full protocol at [rtWIZARD.lji.org](http://rtWIZARD.lji.org)

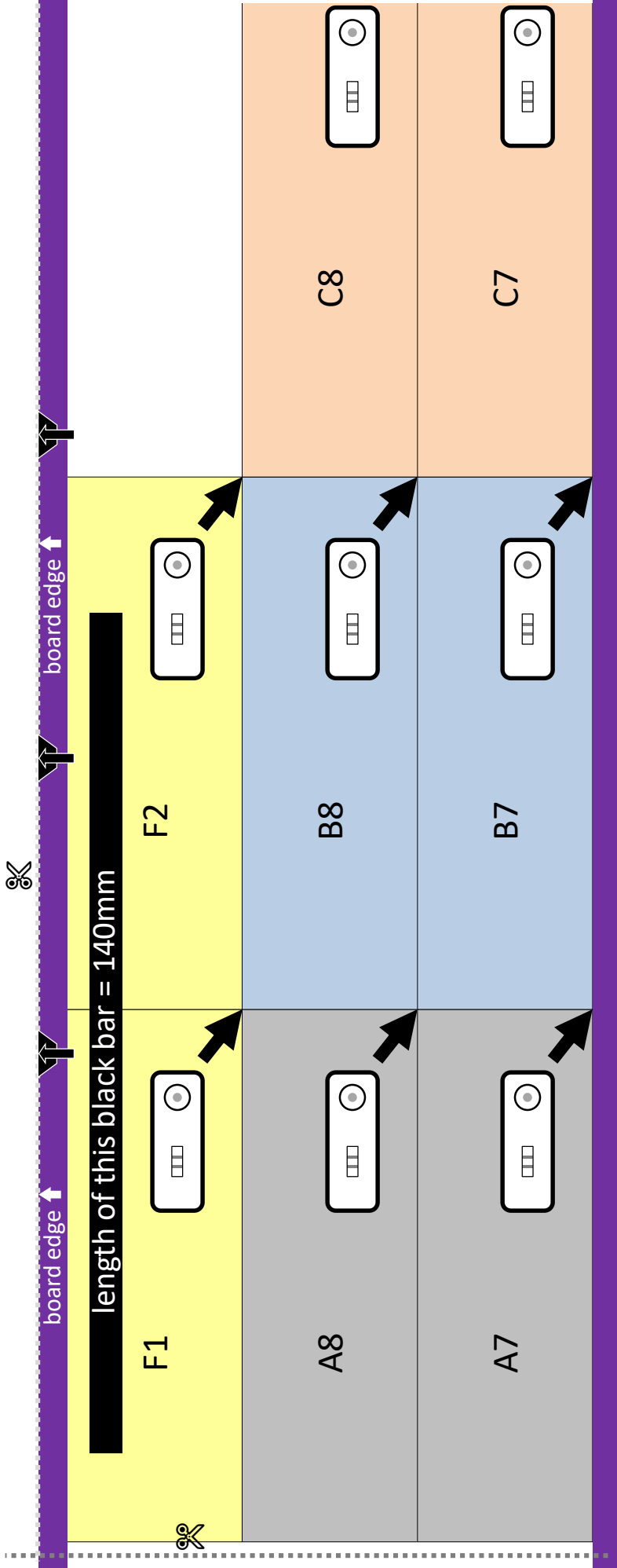

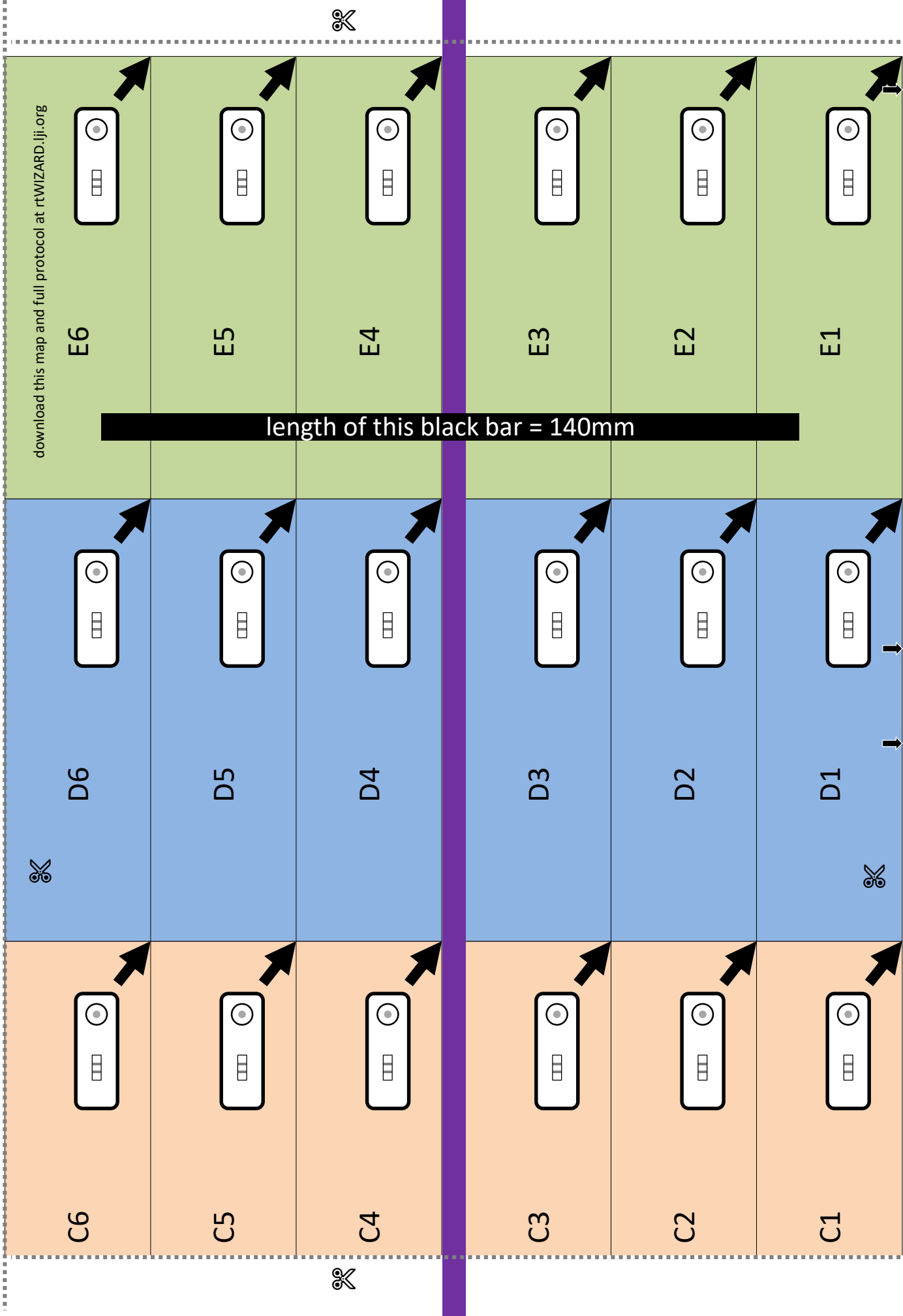

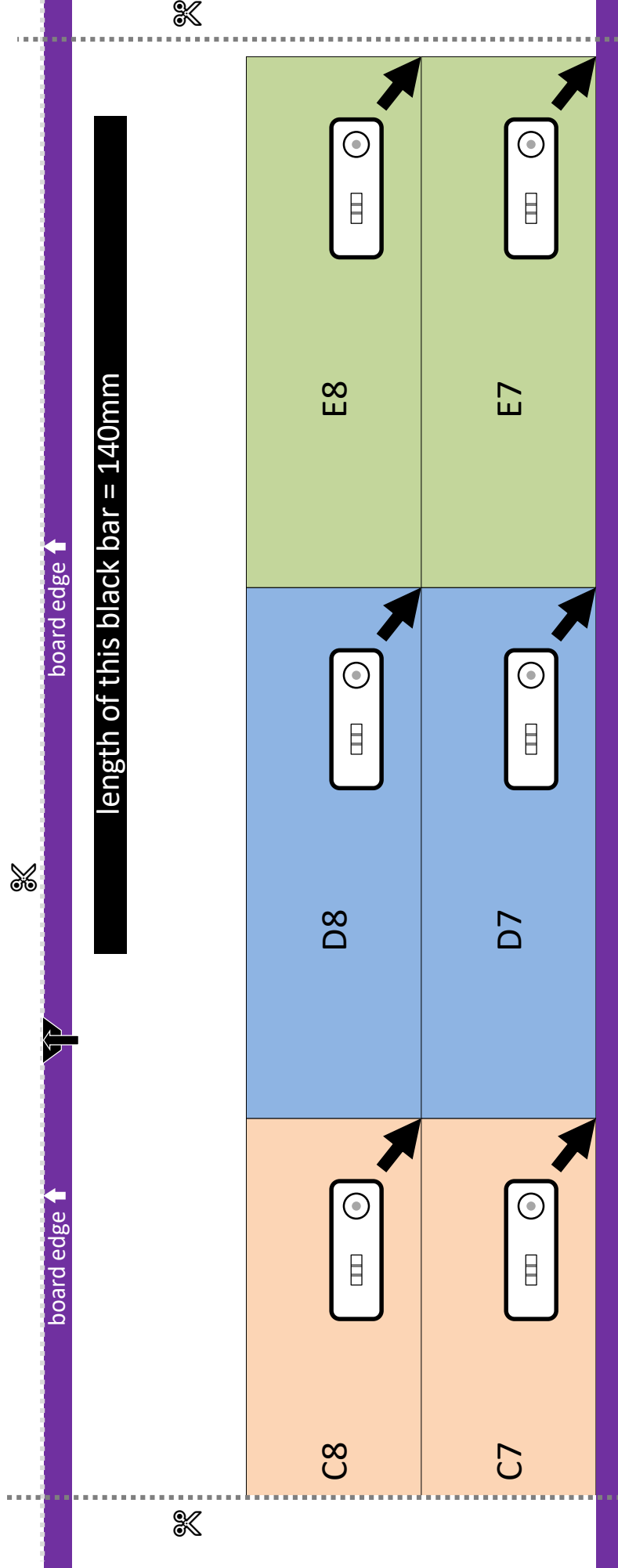

Supplement: S1 Appendix — (PDF) [file pgph.0002625.s002.pdf]

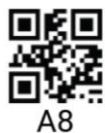

A8

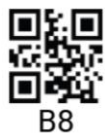

B8

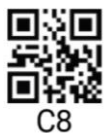

C8

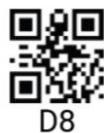

D8

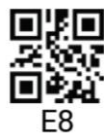

E8

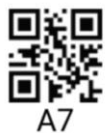

A7

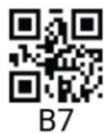

B7

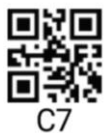

C7

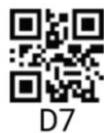

D7

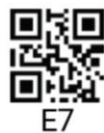

E7

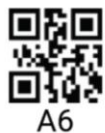

A6

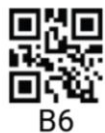

B6

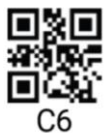

C6

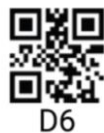

D6

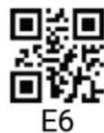

E6

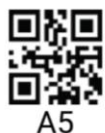

A5

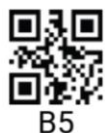

B5

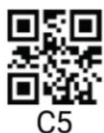

C5

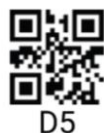

D5

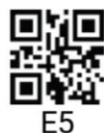

E5

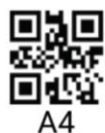

A4

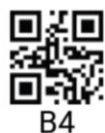

B4

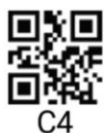

C4

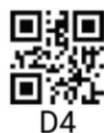

D4

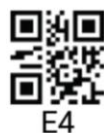

E4

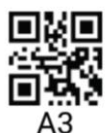

A3

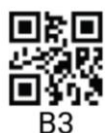

B3

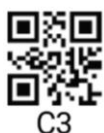

C3

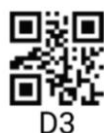

D3

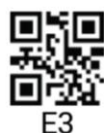

E3

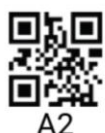

A2

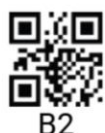

B2

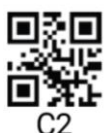

C2

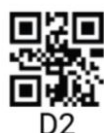

D2

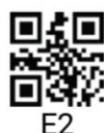

E2

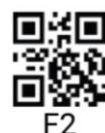

F2

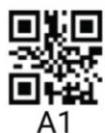

A1

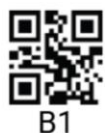

B1

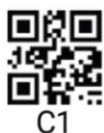

C1

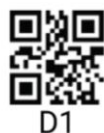

D1

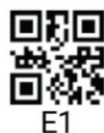

E1

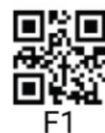

F1

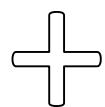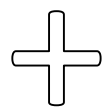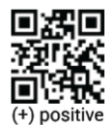

(+) positive

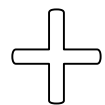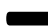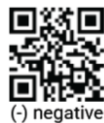

(-) negative

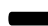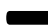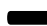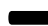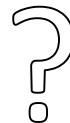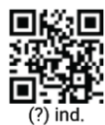

(?) ind.

Supplement: S4 Appendix — (PDF) [file pgph.0002625.s005.pdf]
