# Supplementary material for: Automation for lateral flow rapid tests: Protocol for an open-source fluid handler and applications to dengue and African swine fever tests
Source: PLOS Glob Public Health. 2024 Nov 25;4(11):e0002625. doi: 10.1371/journal.pgph.0002625 (PMC11588214; doi:10.1371/journal.pgph.0002625)
Supplement: S2 Appendix — (PDF) [file pgph.0002625.s003.pdf]

Appendix 2

FOR RESEARCH USE ONLY / SÓLO PARA USO DE INVESTIGACIÓN / POUR USAGE DE RECHERCHE UNIQUEMENT
仅供研究使用 / 研究用のみ / للاستخدام في البحوث فقط

rtWIZARD Manual Plate Record for One-Analyte Test

	Sample ID	Result		Sample ID	Result		Sample ID	Result		Sample ID	Result		Sample ID	Result
F1		+ / - / Ø	F2		+ / - / Ø									
A8		+ / - / Ø	B8		+ / - / Ø	C8		+ / - / Ø	D8		+ / - / Ø	E8		+ / - / Ø
A7		+ / - / Ø	B7		+ / - / Ø	C7		+ / - / Ø	D7		+ / - / Ø	E7		+ / - / Ø
A6		+ / - / Ø	B6		+ / - / Ø	C6		+ / - / Ø	D6		+ / - / Ø	E6		+ / - / Ø
A5		+ / - / Ø	B5		+ / - / Ø	C5		+ / - / Ø	D5		+ / - / Ø	E5		+ / - / Ø
A4		+ / - / Ø	B4		+ / - / Ø	C4		+ / - / Ø	D4		+ / - / Ø	E4		+ / - / Ø
A3		+ / - / Ø	B3		+ / - / Ø	C3		+ / - / Ø	D3		+ / - / Ø	E3		+ / - / Ø
A2		+ / - / Ø	B2		+ / - / Ø	C2		+ / - / Ø	D2		+ / - / Ø	E2		+ / - / Ø
A1		+ / - / Ø	B1		+ / - / Ø	C1		+ / - / Ø	D1		+ / - / Ø	E1		+ / - / Ø

Date: ________________________

Location: ________________________

Ambient temperature: ________________________ Humidity: ________________________

Operator: ________________________

Time fluid handler start: ________________________ Time fluid handler finish: ________________________

Test lot number(s): ________________________
