# Supplementary material for: Automation for lateral flow rapid tests: Protocol for an open-source fluid handler and applications to dengue and African swine fever tests
Source: PLOS Glob Public Health. 2024 Nov 25;4(11):e0002625. doi: 10.1371/journal.pgph.0002625 (PMC11588214; doi:10.1371/journal.pgph.0002625)
Supplement: S3 Appendix — (PDF) [file pgph.0002625.s004.pdf]

**Appendix 3**

**FOR RESEARCH USE ONLY / SÓLO PARA USO DE INVESTIGACIÓN / POUR USAGE DE RECHERCHE UNIQUEMENT / 仅供研究使用 / 研究用のみ / للاستخدام في البحوث فقط**

## rtWIZARD Manual Plate Record for Two-Analyte Test

| | Sample ID | Result 1 | Result 2 | | Sample ID | Result 1 | Result 2 | | Sample ID | Result 1 | Result 2 | | Sample ID | Result 1 | Result 2 | | Sample ID | Result 1 | Result 2 |
|---|---|---|---|---|---|---|---|---|---|---|---|---|---|---|---|---|---|---|---|
| F1 | | + / - / Ø | + / - / Ø | F2 | | + / - / Ø | + / - / Ø | | | | | | | | | | | | |
| A8 | | + / - / Ø | + / - / Ø | B8 | | + / - / Ø | + / - / Ø | C8 | | + / - / Ø | + / - / Ø | D8 | | + / - / Ø | + / - / Ø | E8 | | + / - / Ø | + / - / Ø |
| A7 | | + / - / Ø | + / - / Ø | B7 | | + / - / Ø | + / - / Ø | C7 | | + / - / Ø | + / - / Ø | D7 | | + / - / Ø | + / - / Ø | E7 | | + / - / Ø | + / - / Ø |
| A6 | | + / - / Ø | + / - / Ø | B6 | | + / - / Ø | + / - / Ø | C6 | | + / - / Ø | + / - / Ø | D6 | | + / - / Ø | + / - / Ø | E6 | | + / - / Ø | + / - / Ø |
| A5 | | + / - / Ø | + / - / Ø | B5 | | + / - / Ø | + / - / Ø | C5 | | + / - / Ø | + / - / Ø | D5 | | + / - / Ø | + / - / Ø | E5 | | + / - / Ø | + / - / Ø |
| A4 | | + / - / Ø | + / - / Ø | B4 | | + / - / Ø | + / - / Ø | C4 | | + / - / Ø | + / - / Ø | D4 | | + / - / Ø | + / - / Ø | E4 | | + / - / Ø | + / - / Ø |
| A3 | | + / - / Ø | + / - / Ø | B3 | | + / - / Ø | + / - / Ø | C3 | | + / - / Ø | + / - / Ø | D3 | | + / - / Ø | + / - / Ø | E3 | | + / - / Ø | + / - / Ø |
| A2 | | + / - / Ø | + / - / Ø | B2 | | + / - / Ø | + / - / Ø | C2 | | + / - / Ø | + / - / Ø | D2 | | + / - / Ø | + / - / Ø | E2 | | + / - / Ø | + / - / Ø |
| A1 | | + / - / Ø | + / - / Ø | B1 | | + / - / Ø | + / - / Ø | C1 | | + / - / Ø | + / - / Ø | D1 | | + / - / Ø | + / - / Ø | E1 | | + / - / Ø | + / - / Ø |

Date: ______________________

Location: ______________________

Ambient temperature: ______________________ Humidity: ______________________

Operator: ______________________

Time fluid handler start: ______________________ Time fluid handler finish: ______________________

Test lot number(s): ______________________
