# Supplementary material for: Automation for lateral flow rapid tests: Protocol for an open-source fluid handler and applications to dengue and African swine fever tests
Source: PLOS Glob Public Health. 2024 Nov 25;4(11):e0002625. doi: 10.1371/journal.pgph.0002625 (PMC11588214; doi:10.1371/journal.pgph.0002625)
Supplement: S5 Appendix — (PDF) [file pgph.0002625.s006.pdf]

APPENDIX 5

	Source	Catalog	Quantity	Cost in 2023 (USD)
**SINGLE SOURCE**				
OT-2 lab robot, P300 Single Channel GEN2 Pipette	Opentrons	NA	1	7750
OT-2 Pipette Tips, 300µL, racked	Opentrons	999-00009	9600	550
OT-2 Pipette Tips, 300µL, loose	Opentrons	999-00012	9600	330
**SUBSTITUTABLE**				
Windows laptop with 512MB RAM, 300MB memory, 64-bit	Several	Several	1	200
Corning 3548 well plate	Corning	3548	100	541
LABFISH Microcentrifuge MC-4S (8 tube capacity)	Amazon	B09YR69ZYF	1	60
Pulsar G2319N Inverter Generator	Amazon	B00YFT914I	1	349
Grecell T-500 power station	Amazon	B09ZKT5FQZ	1	357
Tera 1D 2D QR Barcode Scanner	Amazon	B07M68LS2N	1	60
1.1ml Micro serum separator tube	Sarstedt	41.1378.005	100	67
1.3ml Micro Serum tube	Sarstedt	41.1392.105	100	37
1.3ml Micro EDTA tube	Sarstedt	41.1395.105	100	37
2ml centrifuge tube	Several	Several	Several	Several
Avery 94102-WMF10 0.75 x 0.75" labels	Amazon	B0BLJ9S2ZB	2000	35
Winco 12 x 18" Cutting Board	Amazon	B001D3LQVA	1	8
Adoric 0-6" Digital Caliper Measuring Tool	Amazon	B07DFFYCXS	1	9
**DESSICATORS**				
(Chemical) DampRid - several sizes available	Several	Several	Several	Several
(Chemical) Wenko Portable Small Dehumidifier	Amazon	B0B8NDXDBF	1	16
(Battery) ProBreeze PB-04-US Dehumidifier	Amazon	B01DCF0T1Y	1	16
(110V Rechargable) Hornady Dehumidifier 95900	Amazon	B07KCTRVMT	1	28
(110V or 12V) Eva-Dry Dehumidifier EDV-1100	Amazon	B000H0ZDD2	1	40
(110V or 12V) Eva-Dry Mini Dehumidifier EDV-1200	Amazon	B085HHJZRM	1	60
(120V) Caframo SEEKR Stor-Dry Air Circulator	Amazon	B0009L675W	1	91
